# Editorial: Molecular Biology of *Bamboo mosaic Virus*—A Type Member of the Potexvirus Genus

**DOI:** 10.3389/fmicb.2018.00006

**Published:** 2018-01-22

**Authors:** Yau-Heiu Hsu, Ching-Hsiu Tsai, Na-Sheng Lin

**Affiliations:** ^1^Graduate Institute of Biotechnology, National Chung-Hsing University, Taichung, Taiwan; ^2^Institute of Plant and Microbial Biology, Academia Sinica, Taipei, Taiwan

**Keywords:** Bamboo mosaic virus, viral RNA replication, viral trafficking and movement, replicase, viral vector vaccine, insect transmission, host proteins, plant hormone

Bamboo mosaic was first reported in Brazil in 1975, then in Taiwan, Australia, USA, India, China, etc. The causal agent, *Bamboo mosaic virus* (BaMV), is a member of the *Potexvirus* genus of *Flexiviridae*. BaMV can infect at least 12 species in 3 genera of bamboo. Symptoms include chlorotic mosaic on infected leaves and necrotic streaking on shoots and culms. With a high incidence of more than 90% infection in many bamboo plantations, the virus causes great economic loss, particularly in Taiwan, in terms of the quality and quantity of bamboo shoot production, a popular food.

However, study of BaMV did not start extensively until BaMV was identified as a member of the Potexvirus genus at the molecular level, in the early 1990s. Furthermore, the satellite RNA (satRNA) associated with BaMV (satBaMV) was found to be the only satRNA in Potexvirus. After completion of the full-length sequencing of genomic RNA of BaMV and generation of the BaMV infectious cDNA clone, study of BaMV stepped into a new dimension. Since then, BaMV has been extensively studied at the molecular level, covering different both basic and applied research, by a group of researchers in Taiwan. To date, more than 100 BaMV papers have been published in SCI international journals.

Like all potexviruses, BaMV has a flexuous morphology with a single-stranded positive-sense RNA genome. By investigating BaMV, the filamentous structure of flexible viruses was first determined at the near-atomic level by cryo-electron microscopy. This finding solved the mystery of how flexible virus particles maintain structural integrity as mechanical forces deform their structure. In addition, two types of subviral agents, defective RNA and satellite RNA, were found associated with BaMV in the field. Besides studying the viral RNA genomic structure and function, BaMV research has also focused on the virus-host interaction.

In this Research Topic, we summarize the BaMV research with 6 reviews and 4 research articles. To uncover the functional activity of replicase encoded by a positive-sense RNA virus, Meng and Lee describe the enzymatic activities associated with each of the functional domains of the BaMV-encoded replicase, including its unique capping mechanism, which may be conserved across the alphavirus superfamily (Meng and Lee). The authors also detail the interactions between replicase and the host proteins identified in *Nicotiana benthamiana*. Chen et al. cover the location of *cis*-acting elements in the viral RNA and their specific functions, including the recognition of the replicase complex for minus-strand, plus-strand and subgenomic RNA syntheses, viral RNA packaging, and viral movement. Moreover, the authors reveal the host factors that might be involved in delivering BaMV cargoes during intracellular movement, including the delivery of viral RNA to the chloroplast for replication and viral RNA complex to move cell to cell (Cheng). Huang et al. describe the strategies used to identify the host proteins positively and negatively regulating the BaMV RNA replication and viral movements. One of the host proteins reported to be downregulated after BaMV infection is now revealed as carbonic anhydrase (Chen et al.). Nuclear-encoded chloroplast carbonic anhydrase may be involved in the re-initiation step of BaMV RNA replication.

In addition to host factors revealed in replication and movement, the study of BaMV-plant interactions explores a role for abscisic acid (ABA), a plant hormone, in the antiviral silencing pathway that could lead to interference in virus accumulation. Alazem and Lin describe how ABA affects the accumulation of BaMV and other viruses via the gene silencing pathway (Alazem and Lin). BaMV is unique in some isolates carrying satBaMVs, particularly the interfering satBaMV, with crucial roles in modulating BaMV replication and viral symptom development. Lin and Lin summarize the molecular mechanisms underlying the interaction of interfering satBaMV and BaMV (Lin and Lin).

Two papers are related to BaMV ecology. No insect vector for potexviruses has yet been uncovered. Chang et al. investigated the possibility of insect-mediated transmission of BaMV among bamboo clumps and found that two dipteran insects, *Gastrozona fasciventris* and *Atherigona orientalis*, could transmit BaMV to bamboo seedlings. The findings should help in managing BaMV infection by integrating the dipteran insect control. In addition, with decades of collections across a wide geographic area in Asia, Wang et al. have accumulated a sizable number of BaMV and satBaMV isolates for reconstruction of the BaMV and satBaMV phylogeny. The clustering results suggest that the Taiwan Strait was a physical barrier to gene flow in the evolutionary history of both BaMV and satBaMV.

Finally, Chen et al. describe a BaMV-based vector system for peptide presentation. Chimeric BaMV virus expressing the epitope of Japanese encephalitis virus (JEV) could stimulate effective neutralizing antibodies against JEV infection in mice. This study demonstrates an alternative way to produce an effective vaccine candidate against JEV in plants by the BaMV-based vector system.

We thank our colleagues and friends, in total 27 authors, for their valuable contributions to this eBook and cohesive collaboration in the study of BaMV biology in the past 2 decades. We are also grateful to Dr. Anne Simon (University of Maryland), who encouraged us to initiate this Research Topic. We hope the knowledge we have gained from BaMV can serve as the groundwork and biotechnological application for Potexvirus research and RNA research as well.

## Author contributions

All authors listed have made substantial, direct and intellectual contribution to the work, and approved it for publication.

### Conflict of interest statement

The authors declare that the research was conducted in the absence of any commercial or financial relationships that could be construed as a potential conflict of interest.

